# Characterization of *RNF43* frameshift mutations that drive Wnt ligand‐ and R‐spondin‐dependent colon cancer

**DOI:** 10.1002/path.5868

**Published:** 2022-03-04

**Authors:** Daisuke Yamamoto, Hiroko Oshima, Dong Wang, Haruna Takeda, Kenji Kita, Xuelian Lei, Mizuho Nakayama, Kazuhiro Murakami, Takashi Ohama, Hirofumi Takemura, Mutsumi Toyota, Hiromu Suzuki, Noriyuki Inaki, Masanobu Oshima

**Affiliations:** ^1^ Division of Genetics, Cancer Research Institute Kanazawa University Kanazawa Japan; ^2^ Department of Thoracic, Cardiovascular and General Surgery Kanazawa University Kanazawa Japan; ^3^ Department of Gastroenterological Surgery Ishikawa Prefectural Central Hospital Kanazawa Japan; ^4^ WPI Nano‐Life Science Institute (Nano‐LSI) Kanazawa University Kanazawa Japan; ^5^ Laboratory of Molecular Genetics, National Cancer Center Research Institute Tokyo Japan; ^6^ Central Research Resource Branch, Cancer Research Institute Kanazawa University Kanazawa Japan; ^7^ Division of Stem Cell Biology, Cancer Research Institute Kanazawa University Kanazawa Japan; ^8^ Laboratory of Veterinary Pharmacology, Joint Faculty of Veterinary Medicine Yamaguchi University Yamaguchi Japan; ^9^ Department of Molecular Biology Sapporo Medical University School of Medicine Sapporo Japan; ^10^ Department of Gastrointestinal Surgery Kanazawa University Kanazawa Japan

**Keywords:** *RNF43*, serrated pathway, colorectal cancer, organoid, Wnt ligand, PORCN inhibitor

## Abstract

Loss‐of‐function mutations in *RNF43* induce activation of Wnt ligand‐dependent Wnt/β‐catenin signaling through stabilization of the Frizzled receptor, which is often found in microsatellite instability (MSI)‐type colorectal cancer (CRC) that develops from sessile serrated adenomas. However, the mechanism underlying how *RNF43* mutations promote tumorigenesis remains poorly understood. In this study, we established nine human CRC‐derived organoids and found that three organoid lines carried *RNF43* frameshift mutations associated with MSI‐high and *BRAF* ^*V600E*^ mutations, suggesting that these CRCs developed through the serrated pathway. *RNF43* frameshift mutant organoids required both Wnt ligands and R‐spondin for proliferation, indicating that suppression of ZNRF3 and retained RNF43 function by R‐spondin are required to achieve an indispensable level of Wnt activation for tumorigenesis. However, active β‐catenin levels in *RNF43*‐mutant organoids were lower than those in *APC* two‐hit mutant CRC, suggesting a lower threshold for Wnt activation in CRC that developed through the serrated pathway. Interestingly, transplantation of *RNF43*‐mutant organoids with intestinal myofibroblasts accelerated the β‐catenin nuclear accumulation and proliferation of xenograft tumors, indicating a key role of stromal cells in the promotion of the malignant phenotype of *RNF43*‐mutant CRC cells. Sequencing of subcloned organoid cell‐expressed transcripts revealed that two organoid lines carried monoallelic *RNF43 cis*‐mutations, with two *RNF43* frameshift mutations introduced in the same allele and the wild‐type *RNF43* allele remaining, while the other organoid line carried two‐hit biallelic *RNF43 trans*‐mutations. These results suggest that heterozygous *RNF43* frameshift mutations contribute to CRC development via the serrated pathway; however, a second‐hit *RNF43* mutation may be advantageous in tumorigenesis compared with a single‐hit mutation through further activation of Wnt signaling. Finally, treatment with the PORCN inhibitor significantly suppressed *RNF43*‐mutant cell‐derived PDX tumor development. These results suggest a novel mechanism underlying *RNF43* mutation‐associated CRC development and the therapeutic potential of Wnt ligand inhibition against *RNF43*‐mutant CRC. © 2022 The Authors. *The Journal of Pathology* published by John Wiley & Sons Ltd on behalf of The Pathological Society of Great Britain and Ireland.

## Introduction

The Wnt signaling pathway plays a role in the maintenance of the stem cells of adult tissues, which is regulated by the destruction complex, in which GSK3 phosphorylates β‐catenin, leading to the degradation of β‐catenin by the ubiquitin–proteasome pathway [1]. Wnt signaling is also regulated at the receptor level; namely, E3 ligases RNF43 and ZNRF3 downregulate Wnt signaling by ubiquitination of the Wnt receptor Frizzled (FZD), leading to its turnover. Mouse genetic studies have shown that the simultaneous disruption of *Rnf43* and *Znrf3* in the intestine results in Wnt ligand‐independent growth and development of intestinal tumors through Wnt signaling activation [2, 3, 4].

Consistent with this, genetic alterations in *RNF43* are found in approximately 18% of colorectal cancer (CRC) cases [5]. *RNF43* mutations were also identified in cancers in the ovary, stomach, and pancreas [6, 7, 8]. In hereditary serrated polyposis, germline *RNF43* mutations were identified [9], and second‐hit inactivation via loss of heterozygosity (LOH) was also found in tumors [10]. In addition, loss of wild‐type *RNF43* was also reported in ovarian cancer [6]. These results suggest a role for two‐hit *RNF43* mutations in the promotion of tumorigenesis.

Serrated intestinal adenomas are considered precursors of CRC [11]. Notably, *RNF43* mutations in sessile adenomas and serrated pathway‐type CRC were associated with the *BRAF* ^*V600E*^ mutation and CpG island methylator phenotype (CIMP), leading to the methylation of the *MLH1* promoter, which further causes microsatellite instability (MSI) without DNA mismatch repair gene mutations [4, 10, 11, 12, 13, 14, 15]. In addition, *RNF43* mutations were detected in MSI cancers in the stomach, esophagus, and uterine endometrium [16], suggesting that *RNF43* mutations are major drivers of MSI‐type cancer. Furthermore, colitis‐associated colon tumor development was accelerated in *Rnf43* ^−/−^ mice [17], and *in vivo* screening using the *Sleeping Beauty* transposon identified *Rnf43* as a candidate driver of intestinal tumorigenesis [18]. These results, taken together, indicate that an increased level of FZD receptors caused by RNF43 dysfunction drives intestinal tumorigenesis via Wnt signaling activation, particularly in MSI‐CRC through the serrated pathway. Wnt is palmitoleated by porcupine *O*‐acyltransferase (PORCN), which is required for Wnt secretion [19], and a PORCN inhibitor suppressed *RNF43*‐mutant CRC cell growth [12, 20]. Consistent with this, translational research indicates that PORCN inhibition is an effective therapeutic strategy against Wnt ligand‐dependent cancer [19, 21].

Recent studies have identified a link between *RNF43* mutation types and their tumorigenic potential [22, 23, 24]. However, the genetic information for *RNF43* mutations is still not sufficient to understand the mechanism of CRC development. Furthermore, it has been shown that 37% of Wnt ligand‐dependent pancreatic cancer cells require exogenous Wnt ligands, while others utilize endogenous ones [25]. Such cell‐type differences have not been well characterized in CRC.

In the present study, we established human CRC‐derived organoids and found *RNF43* frameshift mutations in CIMP‐high/MSI‐high CRC. Importantly, the *RNF43*‐mutant organoids were dependent on endogenous or exogenous Wnt ligands, and PORCN inhibitors significantly suppressed the organoid proliferation and xenograft tumorigenesis. Furthermore, sequencing of subcloned cell‐derived transcripts suggested that heterozygous monoallelic *RNF43* mutations drive the development of CRC and that second‐hit mutations may be advantageous for tumorigenesis.

## Materials and methods

### Establishment of patient tumor‐derived organoids

Human primary CRC samples were obtained from 50 patients who underwent surgical resection at Ishikawa Prefectural Central Hospital, Japan. All specimens were used for the establishment of organoids and histological analyses. All experiments using human samples were approved by the Human Genome/Gene Analysis Research Ethics Committee of Kanazawa University (2016‐086‐433), and written informed consent was obtained from the patients.

### Organoid culture experiments

The CRC specimens were incubated with 1% collagenase type I (#17100‐017; Life Technologies, Carlsbad, CA, USA) for 30 min, filtered with a 100‐μm‐pore filter, and embedded in growth factor‐reduced (GFR) Matrigel (#356231; Corning, Corning, NY, USA). The organoid culture medium is described in Supplementary materials and methods. Organoids were cultured with 50% of L‐WRN cell‐conditioned medium (CM) containing Wnt3a, R‐spondin, and Noggin [26]. In the absence of WRN‐CM (WRN 0%), 100 ng/ml Noggin (Peprotech, Cranbury, NJ, USA) was added to the medium. For Wnt‐ and R‐spondin dependency experiments, 30 ng/ml human Wnt3a (#5036; R&D Systems, Minneapolis, MN, USA) and/or 1 μg/ml human R‐spondin 3 (#3500; R&D Systems) were added. To examine the growth rate, luciferase activity was analyzed using a Cell Titer‐Glo 3D Cell Viability Assay (#G9682; Promega, Madison, WI, USA). To inhibit Wnt ligand signaling, organoids were cultured with PORCN inhibitor: 100 nm Wnt‐C59 (C59) (S7037; Selleckchem, Houston, TX, USA) or 10 μm IWP2 (a gift from Dr David Virshup). In this study, we established one normal colon tissue‐derived organoid (NC8).

Immortalized intestinal myofibroblasts (LmcMF) have been described previously [27]. LmcMF cells were cultured in 8 ml of DMEM for 48 h, and their conditioned medium was collected. Organoids were cultured with 66.7% LmcMF‐CM. To label organoid cells, Venus and tdTomato cDNAs were subcloned into a pPB‐CAG‐IP PiggyBac transposon vector (a gift from Dr Hitoshi Niwa) and co‐transfected with transposase expression vector using Lipofectamine (Thermo Fisher Scientific, Rockford, IL, USA). Transfected clones were selected by puromycin screening.

### Patient‐derived xenograft (PDX) mice

All animal experiments were performed using the protocol approved by the Committee on Animal Experimentation of Kanazawa University.

Organoid cells were subcutaneously (s.c.) transplanted (1 × 10^5^ cells per site) into immunodeficient SCID Hairless Outbred (SHO) mice (Crlj:SHO‐*Prkdc* ^*scid*^ *Hr* ^*hr*^; Charles River, Yokohama, Japan), and tumors were analyzed at 4 weeks after transplantation (*n* = 4 for each organoid line). For inhibitor treatment, mice were treated with the PORCN inhibitor ETC‐159 (S6616; Selleckchem) at 50 mg/kg per day (per oral) for 5–5.5 weeks after transplantation (*n* = 6–8 sites for each cell line). For co‐transplantation experiments, C45 cells were monocultured (2 × 10^5^ cells per site) or co‐cultured with LmcMF (2 × 10^5^ cells per site for both lines) for 1 day and transplanted s.c. into SHO mice (*n* = 4 for each condition).

### Histology and immunohistochemistry

Tissues were fixed in 4% paraformaldehyde, embedded in paraffin, and cut into 4*‐*μm‐thick sections. The sections were used for H&E or immunohistochemistry. The antibodies used for immunohistochemistry are described in Supplementary materials and methods. Immunostaining signals were visualized using an ImmPACT DAB Substrate Kit (Vector Laboratories, Burlingame, CA, USA).

### Immunofluorescence

Cell proliferation was examined by EdU labeling. Organoids were cultured with 10 μm EdU for 1 h and fixed in 4% PBS–formaldehyde and then permeabilized with 0.1% Triton X‐100. Antibodies are described in Supplementary materials and methods. Fluorescence images were obtained using a TCS SP8 confocal microscope (Leica Microsystems, Wetzlar, Germany).

### Immunoblotting

Organoids were lysed in TNE buffer with Complete Mini protease inhibitor cocktail (Roche Diagnostics, Mannheim, Germany), and 10 μg of the protein samples was separated using 10% SDS‐polyacrylamide gel. Antibodies for immunoblotting are described in Supplementary materials and methods. Relative band intensities were measured using ImageJ software (National Institutes of Health, Bethesda, MD, USA).

### Whole‐exome sequencing

Whole‐exome sequencing was performed using original organoid cells (not subclones). Genomic DNA was extracted using the NucleoSpin Tissue XS (Macherey‐Nagel, Düren, Germany). Paired‐end libraries were prepared using a SureSelect Human All Exon V6 kit (Agilent, Fremont, CA, USA) and sequenced using an Illumina NovaSeq 6000 (outsourced to Takara Bio, Kusatsu, Japan). The generated fastq files were mapped onto human reference genome version GRCh37 (hg19) using the DRAGEN Bio‐IT Platform (Illumina, San Diego, CA, USA). Frameshift mutations, nonsense mutations, and reported oncogenic mutations were examined in *APC*, *CTNNB1*, *RNF43*, *ZNRF3*, *KRAS*, *BRAF*, *TGFBR2*, *ACVR2A*, and *TP53*.

### CIMP analyses

Genomic DNA was modified with sodium bisulfite using an EpiTect Bisulfite Kit (Qiagen, Hilden, Germany). Levels of DNA methylation of classic CIMP markers (MINT1, MINT2, MINT31, MLH1, and CDKN2A) and new CIMP markers (CACNA1G, IGF2, NEUROG1, RUNX3, and SOCS1) were analyzed by bisulfite pyrosequencing as described previously [28, 29].

### MSI analyses

Genomic DNA was extracted, and the MSI analysis was performed according to the Revised Bethesda Guidelines for two mononucleotides (BAT25 and BAT26) and two dinucleotide (D2S123 and D5S346) microsatellite markers (System Biotics, Sagamihara, Japan) [30]. If two or more of these markers showed instability, the sample was diagnosed as MSI‐high.

### Organoid subcloning and *RNF43* genotyping

Organoids were dissociated to single cells by trypsin treatment and single cells were seeded into 96‐well plates at 1 cell per well to obtain subclones. Total RNA was prepared from the subclones, and mRNA was reverse‐transcribed (RT) by SuperScript III (Thermo Fisher Scientific), amplified by polymerase chain reaction (PCR), and subcloned into the plasmid vector with Mighty Cloning Reagent Set (#6027; Takara Bio). PCR primer sequences are provided in Supplementary materials and methods. Subcloned cDNAs were sequenced using an Applied Biosystems 3500 genetic analyzer (Thermo Fisher Scientific).

### Genotyping of *RNF43* codon 370

Genotypes of *RNF43* codon 370 were examined using the TaqMan SNP Genotyping Assay (Applied Biosystems, Waltham, MA, USA). The sequences of TaqMan primers and probes for wild‐type *RNF43* codon 370 (VIC) and p.Rpo370fs (FAM) are provided in Supplementary materials and methods. The PCR results were analyzed by an allele discrimination/SNP system in Mx3000P (Stratagene, La Jolla, CA, USA). The custom *RNF43* p.Pro370fs cDNAs were synthesized to obtain homozygous mutant controls.

### Human gene expression database analyses

Human CRC expression data were downloaded from the Cancer Genome Atlas (TCGA) database (https://www.cancer.gov/about‐nci/organization/ccg/research/structural‐genomics/tcga) [31]. The expression data of CRC carrying *APC*‐deep deletions (*n* = 19) or *RNF43* frameshift mutations at an N‐ter of codon 659 (*n* = 12) were extracted, and upstream regulators were examined using Ingenuity Pathway Analysis (IPA) (Ingenuity Systems, Qiagen; www.ingenuity.com). A Z‐score of ≥2 and a *P* value of <0.05 were considered to indicate activation with statistical significance.

### Transposon insertion site analyses

Transposon mutagenesis screening was previously performed in mice carrying sensitizing driver mutations of CRC [18]. Using the screening data, we analyzed the mutation frequency of *Rnf43*, *Znrf3*, and *Apc* in *Trp53* ^*R172H*^ mouse intestinal tumors, as described in Supplementary materials and methods.

### Statistical analyses

The data were analyzed using two‐sided unpaired *t*‐tests and are presented as the mean ± SD for *in vitro* experiments and the mean ± SEM for *in vivo* analyses. *p* < 0.05 was considered statistically significant.

## Results

### Establishment of human CRC‐derived organoids and PDXs

**Figure 1:**
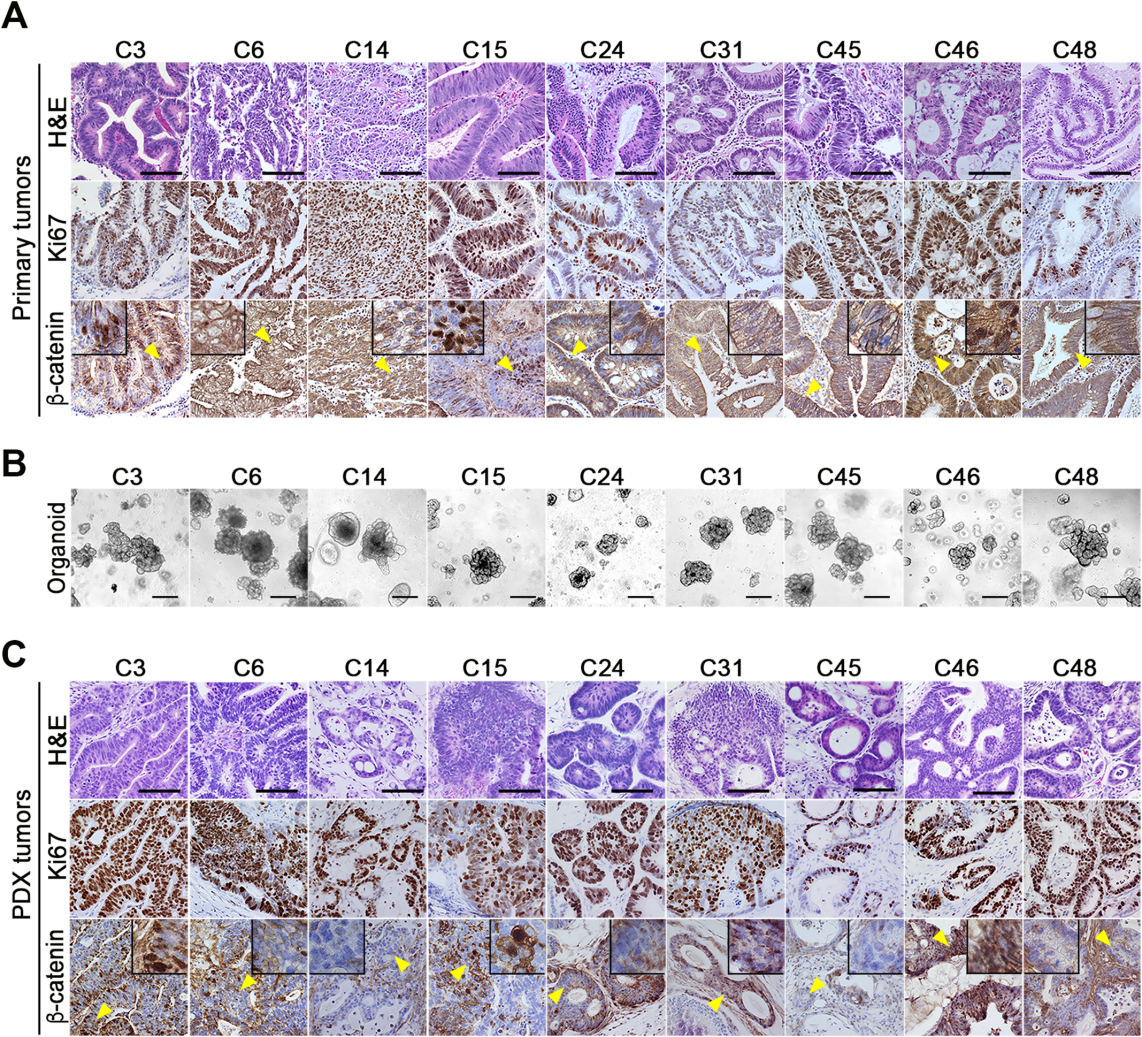
Establishment of human col orectal cancer (CRC)‐derived organoids and patient‐derived xenograft (PDX) models. (A) Representative photographs of histology (H&E) (top) and immunohistochemistry for Ki67 (middle) and β‐catenin (bottom) in the primary CRC tissues of nine patients. Insets are enlarged images of the regions indicated by yellow arrowheads. Bars: 100 μm. (B) Representative photographs of the respective CRC tissue‐derived organoids. Bars: 250 μm. The images are representative of *n* = 3 independent cultures. (C) Representative photographs of histology (H&E) (top) and immunohistochemistry for Ki67 (middle) and β‐catenin (bottom) of the organoid‐transplanted patient‐derived xenograft (PDX) tumors. Insets are enlarged images of the regions indicated by yellow arrowheads. Bars: 100 μm.

Primary CRC tissues were collected from 50 patients, and nine CRC‐derived organoids were successfully established. The clinicopathological characteristics of the nine patients are indicated in supplementary material, Table S1. Histologically, all CRCs, with the exception of C31, were diagnosed as tubular adenocarcinoma; C31 was diagnosed as mucinous adenocarcinoma. Ki67 immunostaining confirmed the increased proliferation of cancer cells in all tissues (Figure 1A). Nuclear accumulation of β‐catenin was detected in C3, C15, and C46 cancer cells, suggesting transcriptional activation of Wnt/β‐catenin signaling.

All established organoid lines showed tubular structures (Figure 1B). When these organoids were s.c. transplanted into SHO mice, all organoids developed tumors with continuous proliferation detected by Ki67 immunostaining (Figure 1C). Histologically, the tubular morphology of the PDX tumors resembled the original primary tumors. Furthermore, β‐catenin nuclear accumulation was detected in the C3, C15, and C46 PDX tumors, indicating that the morphology and Wnt signaling activity of the primary tumors remained in the organoids and PDX tumors.

### *RNF43* mutations in MSI‐type CRC organoids

**Figure 2:**
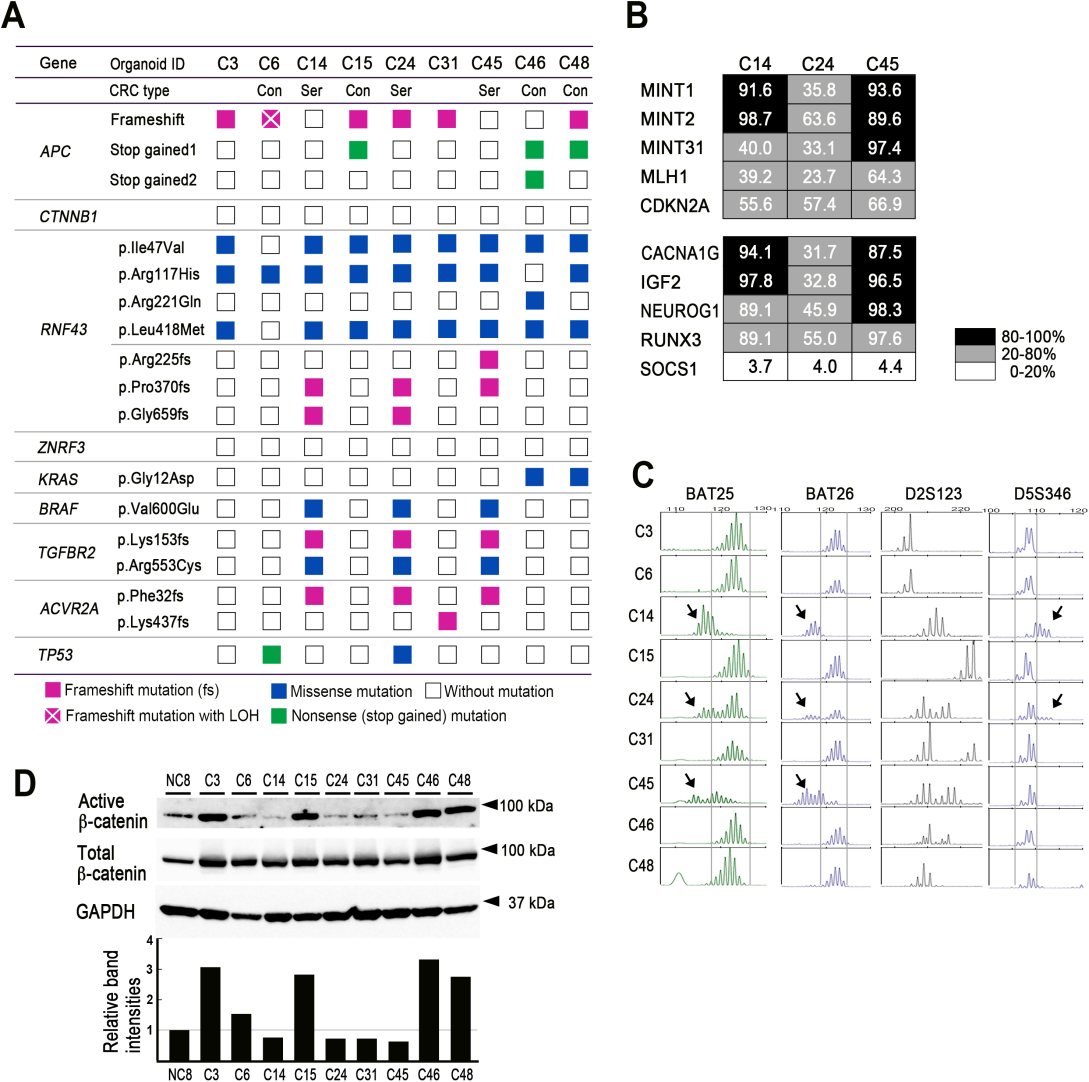
Genetic characterization and β‐catenin activation of CRC‐derived organoid lines. (A) Mutations of the CRC driver genes identified in the respective CRC‐derived organoid lines. The variants of mutations are indicated. Con, conventional pathway CRC; Ser, serrated pathway CRC. (B) The results of CIMP analyses for classic CIMP markers (top) and new CIMP markers (bottom). The methylation levels for each marker in C14, C24, and C45 cells are indicated as a heatmap. (C) The results of microsatellite instability (MSI) marker analyses in BAT25, BAT26, D2S123, and D5S346. Arrows indicate different patterns from other organoid lines. (D) Immunoblotting results for active β‐catenin and total β‐catenin in the respective organoid lines (top). GAPDH was used as an internal control. Normalized band intensities relative to the NC8 level are indicated in the bar graph (bottom).

We performed whole‐exome sequencing of all organoid lines, and mutations in the selected CRC driver genes were analyzed (Figure 2A and supplementary material, Table S2). Possible two‐hit mutations in *APC* were found in C6, C15, C46, and C48 organoids, while *CTNNB1* mutations were not found in any lines. Thus, these CRCs were considered to develop through a conventional pathway (i.e. the adenoma–carcinoma sequence initiated by *APC* two‐hit mutations). Notably, missense mutations in *RNF43* were found in all organoid lines. However, the p.Ile47Val, p.Arg117His, and p.Leu418Met mutations have been shown to maintain the RNF43 function, indicating that they do not contribute to Wnt activation [24]. Notably, C14, C24, and C45 carried frameshift mutations of *RNF43*, i.e. NM_017763.6:c.673del; p.(Arg225Alafs*194), NM_017763.6:c.1109del; p.(Pro370Hisfs*49), and NM_017763.6:c.1976del; p.(Gly659Valfs*41) (hereafter, p.Arg225fs, p.Pro370fs, and p.Gly659fs, respectively). Similar frameshift mutations of *RNF43* were frequently found in serrated adenomas that were often accompanied by CIMP, MSI, and *BRAF* ^*V600E*^ mutations [10, 11, 12, 13, 14, 15]. Pyrosequencing of CIMP markers showed that all classic CIMP markers (MINT1, MINT2, MINT31, MLH1, and CDKN2A) and new CIMP markers (CACNA1G, IGF2, NEUROG1, RUNX3) except for SOCS1 were methylated >20% in C14, C24, and C45 cells, indicating that these cells were CIMP‐high (Figure 2B and supplementary material, Figure S1 and Table S2). Moreover, a microsatellite marker analysis revealed different patterns of BAT25 and BAT26 (mononucleotide repeats) in C14, C24, and C45 cells and D5S346 (dinucleotide repeats) in C14 and C24, indicating an MSI‐high status of these organoid cells (Figure 2C and supplementary material, Table S2). Furthermore, these cells carried *BRAF* ^*V600E*^ mutations and frameshift mutations in *TGFBR2* and *ACVR2A* at polyadenine repeats, which have been reported in MSI CRC cells (Figure 2A) [32, 33]. Taken together, these results suggest that C14, C24, and C45 CRCs developed through the serrated pathway, with *BRAF* mutations as a possible initiation event and subsequent Wnt activation mutation causing a dysplastic phenotype [10, 11, 34]. Loss‐of‐function mutations in *ZNRF3* were not found in any organoids. The molecular characteristics of *RNF43* frameshift mutant organoids are summarized in Table 1.

**Table 1:** Molecular characteristics of *RNF43* frameshift mutant CRC organoids.

	Organoid lines	C14	C24	C45
Genetic/epigenetic status	*RNF43* mutation	p.Pro370fs	p.Pro370fs	p.Arg225fs
		p.Gly659fs	p.Gly659fs	p.Pro370fs
	*BRAF* mutation	p.Val600Glu	p.Val600Glu	p.Val600Glu
	CIMP	High	High	High
	MSI	High	High	High
Organoid growth	Wnt ligand dependency	+	+	+
	Exogenous Wnt ligand	No need*	No need*	Required
	R‐spondin dependency	+	+	+
	Exogenous R‐spondin	Required	Required	Required

* C14 and C24 cells utilize endogenously expressed Wnt ligands.

Interestingly, the immunoblotting results showed significantly higher active β‐catenin levels in CRC organoids with *APC* two‐hit mutations (C15, C46, and C48) than in those with *RNF43* frameshift mutations (C14, C24, and C45) (Figure 2D and supplementary material, Table S2), which is consistent with the nuclear β‐catenin accumulation noted in *APC*‐mutant cancer cells (Figure 1A). Furthermore, an IPA using a public database [29] indicated that Wnt/β‐catenin pathways, such as CTNNB1, WNT1, and lithium chloride, are significantly activated in CRC with *APC* homozygous deletions compared with CRC with *RNF43* truncation mutations (supplementary material, Table S3). These results suggest that the Wnt activation level is lower in serrated pathway CRC cells than in conventional‐type CRC.

### Wnt ligand and R‐spondin dependency of *RNF43* frameshift mutant organoids

**Figure 3:**
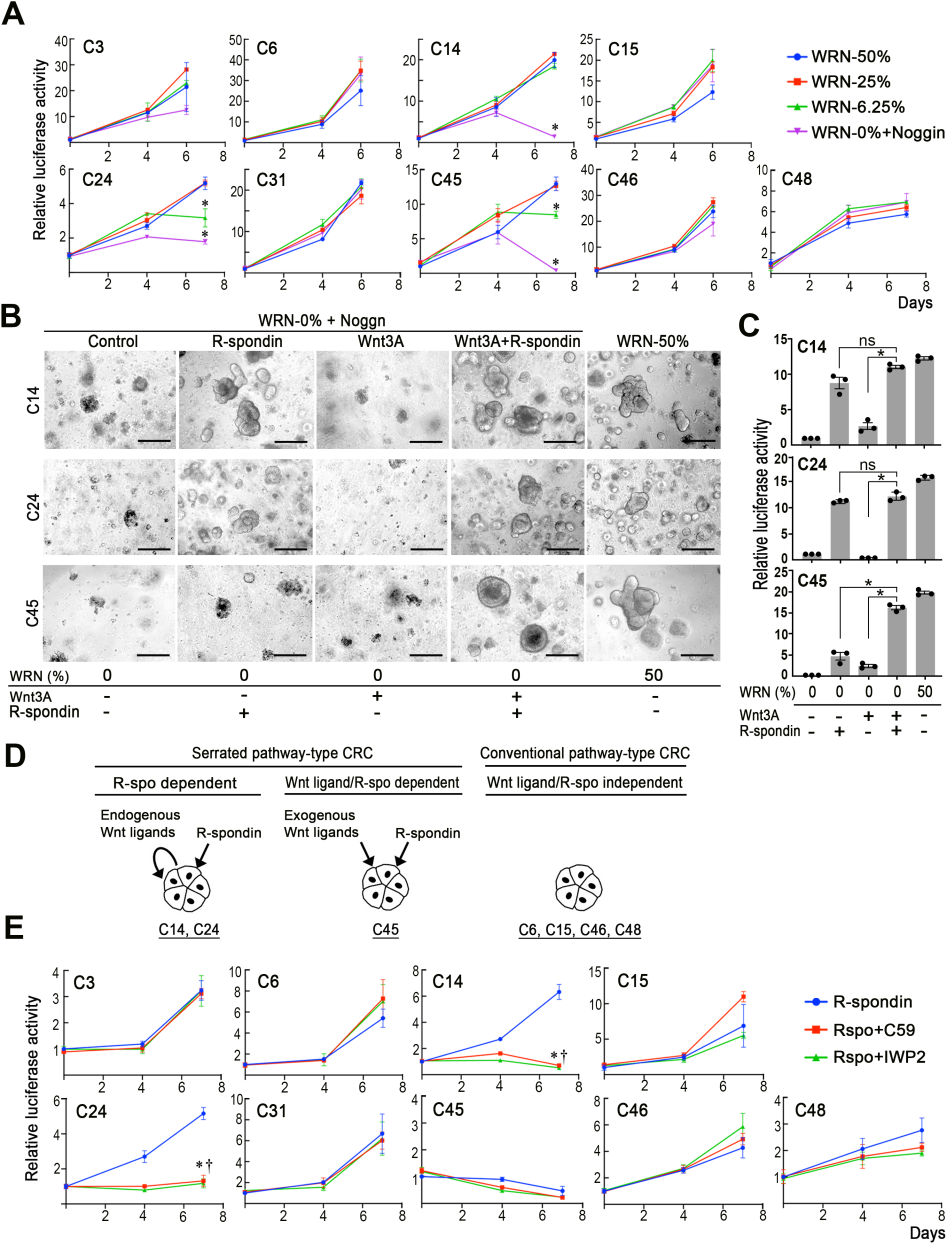
Wnt ligand and R‐spondin dependency of *RNF43* frameshift mutant organoids. (A) Relative cell proliferation, as examined by the luciferase activity of the respective organoids cultured at various concentrations of WRN‐CM, is indicated by line graphs (mean ± SD). A two‐sided *t*‐test was used to calculate statistical significance. *p < 0.05 versus WRN‐50%. (B) Representative photographs of the respective organoids. Culture conditions are indicated below the photographs. Bars: 250 μm. The images are representative of *n* = 3 independent experiments. (C) Relative cell proliferations, as examined by the luciferase activity of organoids cultured under the indicated medium conditions relative to those at control (WRN‐0%), are shown in bar graphs (mean ± SD). Individual data are indicated with dots. A two‐sided *t*‐test was used to calculate statistical significance. *p < 0.05; ns, not significant. (D) Schematic drawing of Wnt ligand and R‐spondin dependency for R‐spondin and endogenous Wnt ligand‐dependent serrated pathway‐type CRC (C14 and C24) (left), R‐spondin and exogenous Wnt ligand‐dependent serrated pathway‐type CRC (C45) (center), and conventional pathway‐type CRC (C6, C15, C46, and C48) (right). (E) Relative cell proliferation, examined by the luciferase activity of the respective organoids cultured in the presence or absence of PORCN inhibitors (C59 or IWP2), is indicated by line graphs (mean ± SD). A two‐sided *t*‐test was used to calculate statistical significance. Asterisks (for R‐spo + C59) and daggers (for R‐spo + IWP2), *p* < 0.05 versus R‐spondin only (no inhibitor) control (blue lines).

We next examined the Wnt ligand dependency of *RNF43* frameshift mutant organoids by reducing the WRN‐conditioned medium (WRN‐CM), which included Wnt3a, R‐spondin, and Noggin. As expected, the number of proliferating cells was significantly decreased in C14, C24, and C45 organoids in the absence of WRN‐CM (Noggin was added to the medium), while other organoids continued proliferating (Figure 3A). Consistent with this, the EdU labeling efficiency was dramatically decreased in *RNF43* frameshift mutant organoids in the WRN‐0% + Noggin condition (supplementary material, Figure S2). Supplementation of WRN‐0% cultures with both recombinant Wnt3a and R‐spondin rescued the proliferation of C14, C24, and C45 organoids to a similar degree as culture with WRN‐50% (Figure 3B,C). Notably, R‐spondin alone also rescued the C14 and C24 organoid proliferation in WRN‐0% + Noggin, while Wnt3a alone did not. Thus, C14 and C24 cells required exogenous R‐spondin but utilized endogenously expressed Wnt ligands (Figure 3D). In contrast, neither Wnt ligand nor R‐spondin rescued C45 cell proliferation, indicating that C45 cells required both exogenous Wnt3a and R‐spondin.

To confirm the Wnt ligand dependency, we treated organoids with the PORCN inhibitors C59 and IWP2 in the presence of R‐spondin under WRN‐0% + Noggin conditions. As expected, both PORCN inhibitors significantly suppressed C14 and C24 cell growth, confirming the need for endogenously expressed Wnt ligand (Figure 3E). In this assay, C45 cells were unable to proliferate because of the absence of exogenous Wnt ligand.

### Proliferation of *RNF43* ‐mutant cells with support from the microenvironment

**Figure 4:**
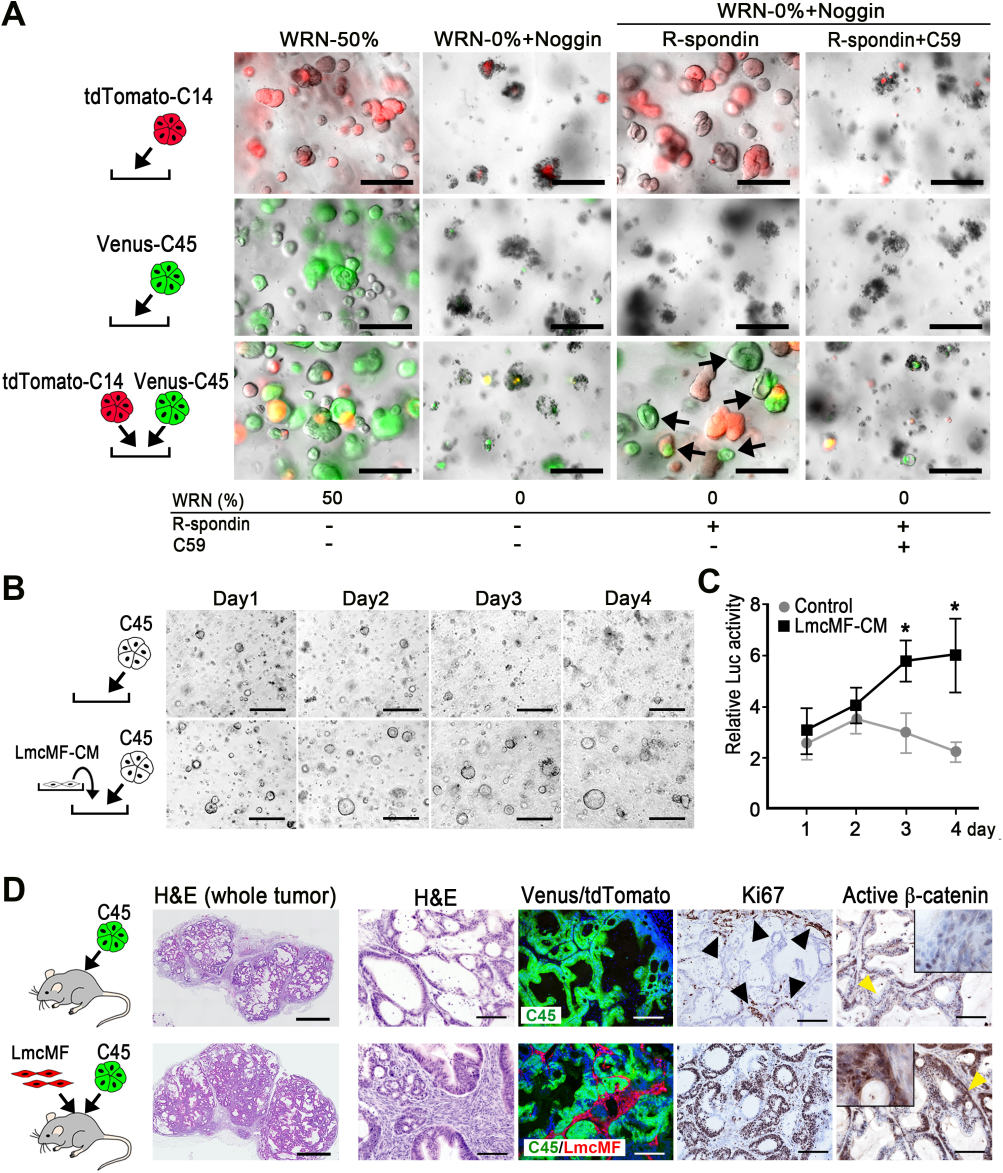
The survival and proliferation of *RNF43* frameshift mutant organoid cells by support from cancer cells and stromal cells. (A) Representative photographs of tdTomato‐labeled C14 (top) and Venus‐labeled C45 (middle) monocultures, and co‐cultures of both organoids (bottom). Culture conditions are indicated below the photographs. Arrows indicate surviving Venus‐labeled C45 cells in a co‐culture with C14 cells under WRN‐0% conditions. Bars: 250 μm. The images are representative of *n* = 3 independent experiments. (B) Representative photographs of C45 organoids cultured under WRN‐0% conditions in the absence (top) or presence of LmcMF cell‐derived conditioned medium (CM) (bottom). Bars: 250 μm. The images are representative of *n* = 3 independent cultures. (C) Relative cell proliferation examined by the luciferase activities of organoids cultured with LmcMF‐CM or control, shown in B, is indicated as a line graph (mean ± SD). A two‐sided *t*‐test was used to calculate statistical significance. *p < 0.05 versus control. (D) Representative photographs of histology (H&E) and immunohistochemistry for Venus/tdTomato (to detect C45 and LmcMF, respectively), Ki67, and active β‐catenin (left to right) of C45 cell‐transplanted tumors (top) and C45 and LmcMF co‐transplanted tumors (bottom). Bars: 1 mm in H&E‐stained whole tumor (left) and 100 μm for other sections. Insets are enlarged images of the regions indicated by yellow arrowheads. Arrowheads indicate Ki67‐positive cells in the marginal zone of C45 tumor tissues. Images are representative of *n* = 4 biologically independent samples.

Lung cancer cells have two distinct subpopulations: one forms a niche providing a Wnt ligand to the other, activating Wnt signaling [35]. It is thus possible that Wnt ligand‐dependent CRC cells utilize the Wnt ligands secreted by other subpopulations. To assess this possibility, C14 and C45 organoid cells were differentially labeled with tdTomato and Venus, respectively, and cultured separately or mixed. We confirmed that R‐spondin rescued the proliferation of C14, but not C45, cells under WRN‐0% + Noggin conditions (Figure 4A). However, C45 cells proliferated under the same culture conditions when co‐cultured with C14 organoids, suggesting that C14‐secreting Wnt ligands activate the Wnt signaling of C45 cells to promote their proliferation.

Stromal cells are also an important source of Wnt ligands and R‐spondin [36]. We therefore cultured C45 organoids in the presence of CM from intestinal myofibroblasts (LmcMF cells) [27] and found that the C45 cells proliferated under WRN‐0% + Noggin conditions, suggesting that LmcMF cells provided sufficient Wnt ligand and R‐spondin to C45 cells (Figure 4B,C). We next co‐transplanted C45 organoids and LmcMF cells into SHO mice s.c. (Figure 4D). Histological analyses indicated that a fibroblast‐rich microenvironment was generated by tdTomato‐labeled LmcMF cells in co‐transplanted tumors (Figure 4D). Ki67‐positive proliferating cells were found only in the marginal zone of C45‐alone tumors (Figure 4D, arrowheads), while proliferation was found in the whole tumor area of co‐transplanted tumors. Furthermore, clear nuclear accumulation of β‐catenin was detected in C45 tumor cells when co‐transplanted with LmcMF cells, which was rarely found in C45‐alone tumors. These results suggest that cancer‐associated fibroblasts contribute to malignant phenotypes of *RNF43*‐mutant CRC by expressing Wnt ligands and R‐spondin.

### Characterization of *RNF43* frameshift mutation in CRC cells

**Figure 5:**
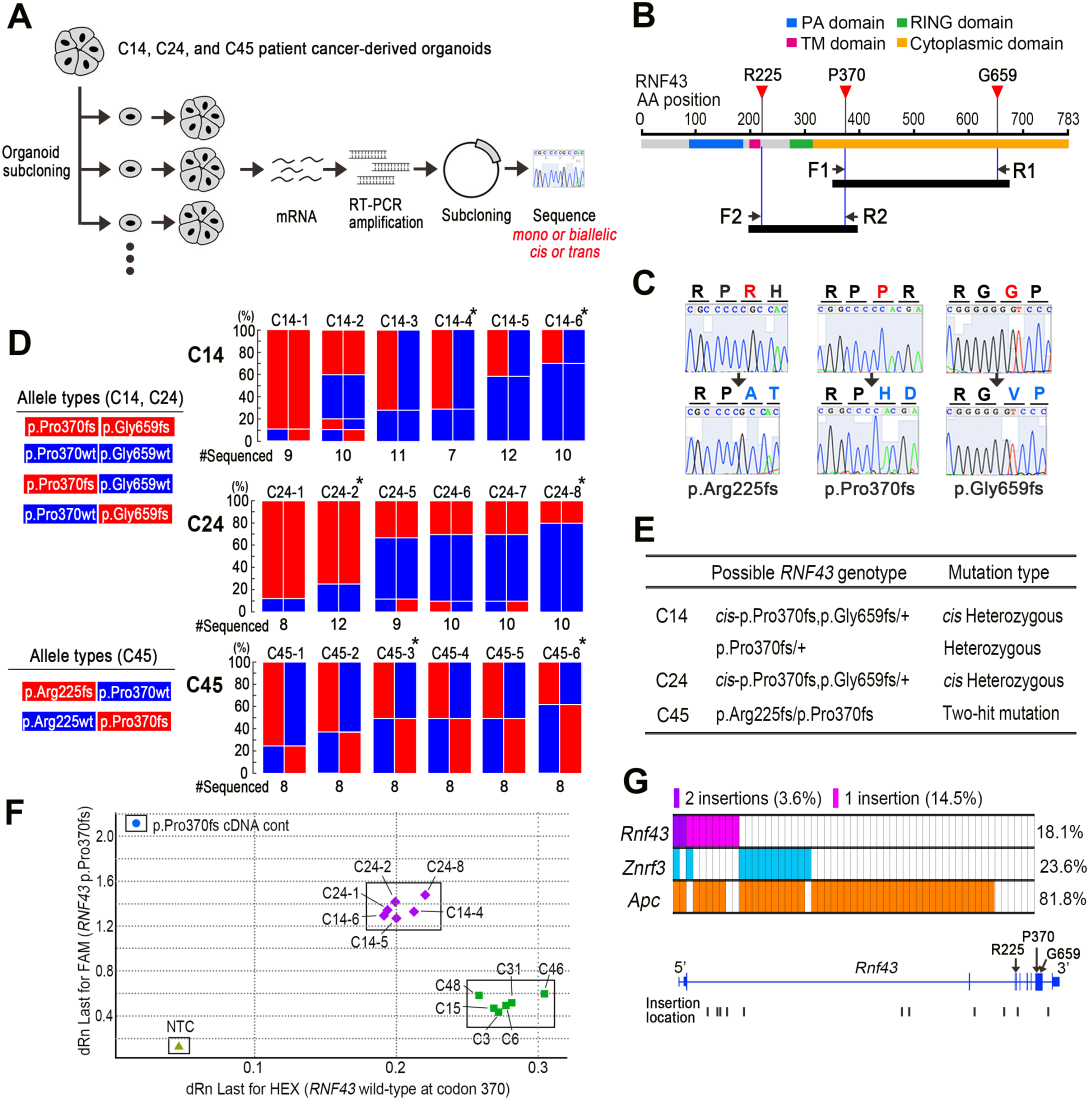
Characterization of *RNF43* mutations in CRC cells. (A) Schematic drawing of the experimental strategy. C14, C24, and C45 *RNF43* frameshift mutant organoids were subjected to subcloning (*n* = 6 subclones for each line); *RNF43* mRNAs were amplified by RT‐PCR, and cDNAs were subcloned to the plasmid (*n* = 7–12 cDNAs for each subclone). *RNF43* mutation patterns were then examined by sequencing. (B) The RT‐PCR strategy to examine *cis* or *trans* mutations of p.Pro370fs and p.Gly659fs by the primer F1 and R1, and p.Arg225fs and p.Pro370fs by the primer F2 and R2. Arrows indicate the locations of the respective primers. Closed bars indicate expected amplified cDNA fragments by the primers. (C) Representative sequencing results of wild‐type (top) and mutant *RNF43* (bottom) for p.Arg225fs (left), p.Pro370fs (center), and p.Gly659fs (right) positions. Note that the mutations cause amino acid changes (blue). (D) Schematic drawing of *RNF43* allele types identified by sequencing in the C14 and C24 subclones (left, top) and C45 subclones (left, bottom). The frequencies of the respective allele types in each subclone are indicated as color bars for C14 (right, top), C24 (right, middle), and C45 (right, bottom). Asterisks indicate subclones used for *in vitro* and xenograft drug dosing experiments (Figure 6 and supplementary material, Figures S3 and S4). The numbers indicated below the bar graphs indicate the total number of independently amplified and sequenced cDNAs. (E) List of possible *RNF43* genotypes of the parental organoid lines. (F) The results of TaqMan SNP genotyping at *RNF43* codon 370 (wild‐type: HEX versus p.Pro370fs: FAM) for C14 and C24 subclones (purple diamonds) and conventional pathway‐type CRC organoids (green squares) are shown as a scatter plot with *RNF43* pPro370fs cDNA control (blue circle) and no template control (NTC) (light‐green triangle). (G) Top: the frequencies of transposon insertions in the *Rnf43*, *Znrf3*, and *Apc* genes in SB‐induced intestinal tumors. Bottom: the locations of transposon insertions in the *Rnf43* gene. Exons including R225, P370, and G659 are indicated by arrows.

C14, C24, and C45 organoid cells carried two *RNF43* frameshift mutations: p.Pro370fs and p.Gly659fs in C14 and C24, and p.Arg225fs and p.Pro370fs in C45. To examine whether these mutations were heterozygous (monoallelic) or biallelic – i.e. *cis* or *trans* mutations – we performed subcloning of the parental organoid lines and examined the mutation types in subclones via the amplification of *RNF43* mRNA by RT‐PCR followed by cDNA sequencing (Figure 5A). PCR primers were designed to amplify cDNA fragments, including two mutation sites at both ends (Figure 5B). cDNA sequencing indicated a 1‐bp deletion in (C)_5_ for p.Arg225fs and p.Pro370fs and in (G)_7_ for p.Gly659fs, resulting in amino acid changes (Figure 5C).

Before sequencing, we confirmed the Wnt ligand and R‐spondin dependency of the organoid subclones (supplementary material, Figure S3A). Furthermore, *AXIN2* and *ZNRF3* expression was significantly decreased in C14‐, C24‐, and C45‐derived subclones compared with *APC*‐mutated conventional‐type CRC organoids (supplementary material, Figure S3B,C), which was a characteristic of CIMP‐high/MSI‐high serrated CRC [12, 22, 37].

*RNF43* mutations in the cDNA fragments were then examined by sequencing, and a majority of C24 subclones showed *cis*‐p.Pro370fs, p.Gly659fs mutations or wild‐type *RNF43*, indicating that the *RNF43* genotype of the parental C24 organoids was an *RNF43* ^*cis*‐p.Pro370fs,p.Gly659fs/+^ heterozygous mutation (Figure 5D, middle; 5E). In contrast, C45 subclones showed either simple p.Arg225fs or p.Pro370fs mutations, indicating that the *RNF43* genotype of parental C45 cells was *RNF43* ^p.Arg225fs/p.Pro370fs^, a two‐hit biallelic mutation (Figure 5D, bottom; 5E). In contrast, C14 subclones showed at least two different genotypes (i.e. *RNF43* ^*cis*‐p.Pro370fs,p.Gly659fs/+^ and *RNF43* ^p.Pro370fs/+^) (Figure 5D, top; 5E). It is possible that C14 parental organoids carried genetic heterogeneity and that different genotype cells were subcloned, although this point remains to be examined.

Importantly, allele‐specific genomic PCR indicated that C14 and C24 subclones possessed both *RNF43* codon 370 wild‐type and mutant genes with the same copy number, while conventional pathway‐type CRC organoids had only wild‐type *RNF43* (Figure 5F). These results exclude the possibility of loss of wild‐type *RNF43* by LOH in C14 and C24 cells. Interestingly, the mutant allele frequency increased to approximately 90% in C14‐1 and C24‐1 subclones. The wild‐type *RNF43* gene may have been lost by LOH during the subcloning process, allowing LOH cells to become dominant because of a possible growth advantage.

We further examined the data previously obtained by transposon mutagenesis screening in mouse intestine with the *Trp53* R172H genetic background [18]. The frequencies of *Rnf43*, *Znrf3*, and *Apc* mutations by transposon insertions in intestinal tumors are indicated in Figure 5G (top). Single insertions in *Rnf43* were found in 14.5% of tumors, and two cases did not carry mutations in *Apc* and *Znrf3*. Most transposon insertions were found in introns 2 and 3, resulting in truncation at the N‐terminal of RNF43 (Figure 5G, bottom). These results support the idea that a single‐hit *RNF43* mutation contributes to intestinal tumorigenesis.

### Suppression of *RNF43* ‐mutant PDX tumors by a PORCN inhibitor

**Figure 6:**
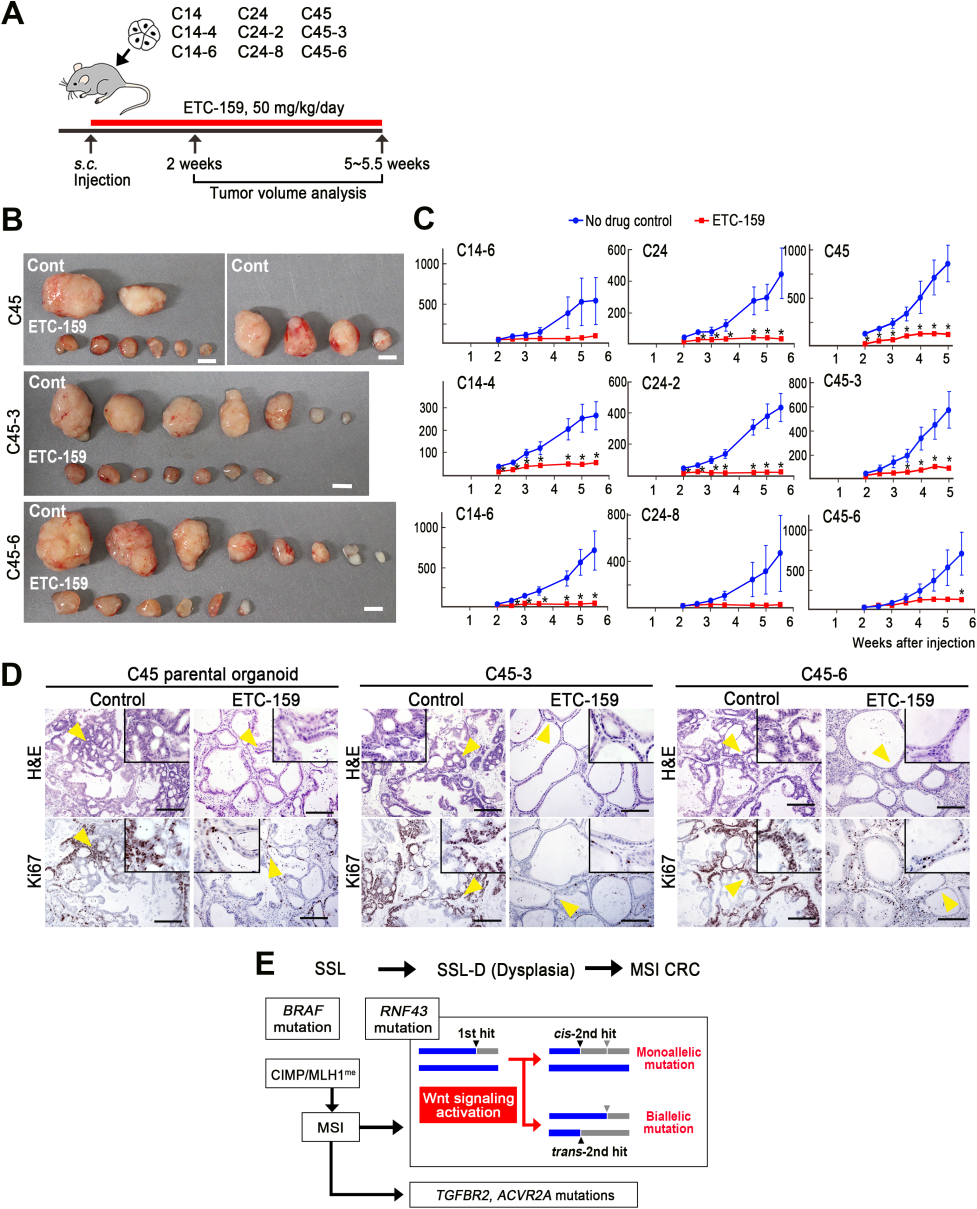
Suppression of *RNF43* frameshift mutant PDX tumor development by a PORCN inhibitor. (A) Schematic drawing of the strategy for the drug dosing experiment. The red bar indicates the PORCN inhibitor (ETC‐159) treatment period. (B) Representative photographs of PDX tumors of C45 parental organoids and two subclones (C45‐3 and C45‐6) developed in no‐drug control mice (top) and ETC‐159‐treated mice (bottom). Bars: 5 mm. Photographs of PDX tumors of C14, C24, and subclones are shown in supplementary material, Figure S4A. (C) Tumor volume changes of the control (blue) and ETC‐159‐treated PDX mice (red) are indicated as line graphs (mean ± SEM). Transplanted organoid clone numbers are indicated. A two‐sided *t*‐test was used to calculate statistical significance.*p < 0.05 versus control. (D) Representative histology photographs of PDX tumors developed in the control (left) and ETC‐159‐treated mice (right) for C45 parental organoids and C45‐3 and C45‐6 subclones (left to right). H&E (top) and immunohistochemistry for Ki67 (bottom). Insets are enlarged images of the regions indicated by yellow arrowheads. Bars: 200 μm. The images are representative of *n* = 6–8 biologically independent experiments. Histology results for C14, C24, and subclones are shown in supplementary material, Figure S4B. (E) Schematic illustration of serrated pathway CRC development. SSL, sessile serrated lesion; SSL‐D, SSL with dysplasia; CIMP, CpG island methylator phenotype; MLH1^me^, MLH1 gene promoter hypermethylation.

Finally, we examined whether or not a PORCN inhibitor suppresses the tumor development of *RNF43* frameshift mutant cells in PDX models. C14, C24, and C45 parental and subclone organoids were transplanted s.c. into SHO mice, and mice were treated with the PORCN inhibitor ETC‐159 (Figure 6A). Importantly, ETC‐159 treatment nearly completely suppressed the tumor development in all PDX model mice (Figure 6B,C and supplementary material, Figure S4A). Histological analyses indicated that ETC‐159 treatment resulted in decreased branching and enlarged glands with fewer dysplastic features and significantly decreased proliferation (Figure 6D and supplementary material, Figure S4B). It is possible that cancer cells were differentiated by PORCN inhibition‐induced suppression of Wnt signaling, as previously reported [21].

## Discussion

Serrated polyps with MSI are reportedly associated with a predisposition to progress to CRC [10, 11]. Moreover, *RNF43* mutations were frequently found in sessile serrated lesions (SSLs) and MSI‐type CRC, suggesting that *RNF43* mutation drives CRC development through the serrated pathway (Figure 6E). Consistently, we found *RNF43* frameshift mutations in CIMP‐high and MSI‐high CRC‐derived organoids, which were associated with *BRAF* ^V600E^ mutations.

Interestingly, the level of active β‐catenin in *RNF43*‐mutant CRC was significantly lower than that in *APC* two‐hit CRC, and nuclear accumulation of β‐catenin was found only in *APC*‐mutant CRC. These results suggest that the threshold of Wnt activation for serrated pathway tumorigenesis is lower than that via the conventional pathway. Consistently, it has been reported that Wnt activation is greatly reduced in MSI‐high hypermutated CRCs relative to non‐hypermutated CRCs [37]. It is possible that a high level of Wnt activation is needed for the expansion of intestinal stem/progenitor cells to form adenomatous polyps as an initial event of the conventional pathway, as found in *Apc* ^*Δ716*^ knockout mice [38]. Although adenomatous proliferation was found in *Rnf43* ^*−/−*^ *Znrf3* ^*−/−*^ compound mouse intestine [2, 3], it was not observed in *Rnf43* ^*−/−*^ simple mutant mice [17]. These results suggest that the degree of Wnt activation induced by *RNF43* mutation is not sufficient for the initiation of conventional‐type CRC but can contribute to serrated pathway tumorigenesis.

In the present study, all *RNF43* frameshift mutant CRC cells proliferated in an R‐spondin‐dependent manner. It is possible that suppression of ZNRF3 function and retention of RNF43 function by R‐spondin is required to achieve the level of Wnt signaling necessary for tumorigenesis in *RNF43*‐mutant cells.

Loss of wild‐type *RNF43* by LOH or second‐hit somatic mutations has been reported in ovarian tumors and serrated colon tumors [6, 10], suggesting the need for two‐hit inactivation of *RNF43* for tumorigenesis. However, the present results showed heterozygous monoallelic *RNF43* mutations in C14 and C24 cells, indicating that biallelic inactivation of *RNF43* is not necessarily required for serrated pathway tumor development. In contrast, we also found two‐hit biallelic *trans*‐mutations in *RNF43* in C45 cells, suggesting that two‐hit mutations of *RNF43* are advantageous for CRC development, possibly through an increase in the Wnt activation level (Figure 6E). Furthermore, *cis* mutations in *RNF43* for p.Pro370fs and p.Gly659fs were found in the same allele. A recent analysis indicated that the most common mutation of *RNF43*, p.Gly659fs, modestly compromises the RNF43 function [24], and N‐terminal truncation mutations are more effective at increasing Wnt signaling activity than C‐terminal mutations [23]. Therefore, the p.Gly659fs mutation may have been introduced at an early stage of tumorigenesis in C14 and C24 with a slight increase in Wnt signaling. An additional N‐terminal p.Pro370fs mutation was then introduced at the same allele, which may have facilitated tumor promotion by increasing the Wnt activity (Figure 6E).

Importantly, co‐transplantation of *RNF43* frameshift mutant CRC cells with myofibroblasts increased the β‐catenin nuclear accumulation and cell proliferation of PDX tumors, indicating that the malignant phenotype of *RNF43*‐mutant cells is highly dependent on stromal cells. Several studies have demonstrated the role of Wnt signaling in metastasis; namely, Wnt activation promotes the epithelial–mesenchymal transition program by increasing the nuclear Smai2 expression [39], and liver metastasis of colon cancer cells is accelerated by the nuclear accumulation of β‐catenin and FOXO3 [40]. Accordingly, it is possible that metastatic niche cells play a key role in the proliferation of disseminated *RNF43*‐mutant CRC cells through activation of Wnt signaling. In this study, we demonstrated the significant suppression of *RNF43*‐mutant PDX tumors by ETC‐159 treatment. Thus, it is important to further examine whether or not the development of metastatic foci by *RNF43*‐mutant cells can be suppressed by PORCN inhibitors as a future clinical strategy.

In conclusion, we established *RNF43* frameshift CRC‐derived organoids. The Wnt/β‐catenin activation level of *RNF43*‐mutant serrated pathway CRC was lower than that of conventional pathway‐type CRC. Furthermore, heterozygous *RNF43* frameshift mutation may contribute to tumorigenesis through the serrated pathway. However, second‐hit *RNF43* mutations may facilitate CRC development by increasing the Wnt activation level. Finally, PORCN inhibition significantly suppressed PDX tumor development of *RNF43*‐mutant CRC cells, suggesting that PORCN inhibitors are an effective therapeutic strategy against *RNF43*‐mutant CRC development and metastasis.

## Author contributions statement

HO and MO designed the study and planned the experiments. DY, HO and KK were involved in organoid preparation and performed immunohistochemistry. DY, DW, XL, MN and KM were involved in *in vitro* and *in vivo* experiments. MT and HS were involved in CIMP analysis. HaT examined the transposon data. TO provided cancer‐associated fibroblasts. HiT, NI and MO supervised the project. All of the authors were involved in writing the paper and had final approval of the submitted version.

## Supporting information


Supplementary materials and methods

**Figure S1.** The results of CIMP analysis for *RNF43* frameshift mutant organoid cells
**Figure S2.** Wnt ligand and R‐spondin dependency of *RNF43* frameshift mutant organoid cells
**Figure S3.** Biological characteristics of *RNF43* frameshift mutant CRC cells
**Figure S4.** Suppression of *RNF43* frameshift mutant tumor development by PORCN inhibitor
**Table S1**. Clinicopathological characteristics of CRC patients
**Table S2.** Mutation variants and characteristics of CRC organoids
**Table S3**. Activated upstream regulators in *APC*‐deleted CRCs compared with *RNF43* truncation mutation CRCs (IPA)

## References

[1] Nusse R , Clevers H . Wnt/β‐catenin signaling, disease, and emerging therapeutic modalities. Cell 2017; 169: 985–999. 28575679 10.1016/j.cell.2017.05.016

[2] Koo BK , Spit M , Jordens I , *et al*. Tumour suppressor RNF43 is a stem‐cell E3 ligase that induces endocytosis of Wnt receptors. Nature 2012; 488: 665–669. 22895187 10.1038/nature11308

[3] Koo BK , van Es JH , van den Born M , *et al*. Porcupine inhibitor suppresses paracrine Wnt‐driven growth of *Rnf43;Znrf3*‐mutant neoplasia. Proc Natl Acad Sci U S A 2015; 112: 7548–7550. 26023187 10.1073/pnas.1508113112 PMC4475934

[4] Lannagan TRM , Le YK , Wang T , *et al*. Genetic editing of colonic organoids provides a molecularly distinct and orthotopic preclinical model of serrated carcinogenesis. Gut 2019; 68: 684–692. 29666172 10.1136/gutjnl-2017-315920 PMC6192855

[5] Giannakis M , Hodis E , Mu XJ , *et al*. *RNF43* is frequently mutated in colorectal and endometrial cancers. Nat Genet 2014; 46: 1264–1266. 25344691 10.1038/ng.3127 PMC4283570

[6] Ryland GL , Hunter SM , Doyle MA , *et al*. *RNF43* is a tumour suppressor gene mutated in mucinous tumours of the ovary. J Pathol 2013; 229: 469–476. 23096461 10.1002/path.4134

[7] Wang K , Yuen ST , Xu J , *et al*. Whole‐genome sequencing and comprehensive molecular profiling identify new driver mutations in gastric cancer. Nat Genet 2014; 46: 573–582. 24816253 10.1038/ng.2983

[8] Jiang X , Hao HX , Growney JD , *et al*. Inactivating mutations of *RNF43* confer Wnt dependency in pancreatic ductal adenocarcinoma. Proc Natl Acad Sci U S A 2013; 110: 12649–12654. 23847203 10.1073/pnas.1307218110 PMC3732970

[9] Taupin D , Lam W , Rangiah D , *et al*. A deleterious *RNF43* germline mutation in a severely affected serrated polyposis kindred. Hum Genome Var 2015; 2: 15013. 27081527 10.1038/hgv.2015.13 PMC4785559

[10] Yan HHN , Lai JCW , Ho SL , *et al*. RNF43 germline and somatic mutation in serrated neoplasia pathway and its association with BRAF mutation. Gut 2017; 66: 1645–1656. 27329244 10.1136/gutjnl-2016-311849

[11] Crockett SD , Nagtegaal ID . Terminology, molecular features, epidemiology, and management of serrated colorectal neoplasia. Gastroenterology 2019; 157: 949–966.e4. 31323292 10.1053/j.gastro.2019.06.041

[12] Bond CE , McKeone DM , Kalimutho M , *et al*. *RNF43* and *ZNRF3* are commonly altered in serrated pathway colorectal tumorigenesis. Oncotarget 2016; 7: 70589–70600. 27661107 10.18632/oncotarget.12130 PMC5342576

[13] Tsai JH , Liau JY , Yuan CT , *et al*. *RNF43* is an early and specific mutated gene in the serrated pathway, with increased frequency in traditional serrated adenoma and its associated malignancy. Am J Surg Pathol 2016; 40: 1352–1359. 27305845 10.1097/PAS.0000000000000664

[14] Sekine S , Yamashita S , Tanabe T , *et al*. Frequent *PTPRK–RSPO3* fusions and *RNF43* mutations in colorectal traditional serrated adenoma. J Pathol 2016; 239: 133–138. 26924569 10.1002/path.4709

[15] Kleeman SO , Koelzer VH , Jones HJ , *et al*. Exploiting differential Wnt target gene expression to generate a molecular biomarker for colorectal cancer stratification. Gut 2020; 69: 1092–1103. 31563876 10.1136/gutjnl-2019-319126 PMC7212029

[16] Sanchez‐Vega F , Mina M , Armenia J , *et al*. Oncogenic signaling pathways in The Cancer Genome Atlas. Cell 2018; 173: 321–337.e10. 29625050 10.1016/j.cell.2018.03.035 PMC6070353

[17] Eto T , Miyake K , Nosho K , *et al*. Impact of loss‐of‐function mutations at the *RNF43* locus on colorectal cancer development and progression. J Pathol 2018; 245: 445–455. 29756208 10.1002/path.5098

[18] Takeda H , Wei Z , Koso H , *et al*. Transposon mutagenesis identifies genes and evolutionary forces driving gastrointestinal tract tumor progression. Nat Genet 2015; 47: 142–150. 25559195 10.1038/ng.3175

[19] Zhong Z , Virshup DM . Wnt signaling and drug resistance in cancer. Mol Pharmacol 2020; 97: 72–89. 31787618 10.1124/mol.119.117978

[20] van de Wetering M , Francies HE , Francis JM , *et al*. Prospective derivation of a living organoid biobank of colorectal cancer patients. Cell 2015; 161: 933–945. 25957691 10.1016/j.cell.2015.03.053 PMC6428276

[21] Madan B , Ke Z , Harmston N , *et al*. Wnt addiction of genetically defined cancers reversed by PORCN inhibition. Oncogene 2016; 35: 2197–2207. 26257057 10.1038/onc.2015.280 PMC4650263

[22] Tu J , Park S , Yu W , *et al*. The most common RNF43 mutant G659Vfs*41 is fully functional in inhibiting Wnt signaling and unlikely to play a role in tumorigenesis. Sci Rep 2019; 9: 18557. 31811196 10.1038/s41598-019-54931-3 PMC6898356

[23] Li S , Lavrijsen M , Bakker A , *et al*. Commonly observed *RNF43* mutations retain functionality in attenuating Wnt/β‐catenin signaling and unlikely confer Wnt‐dependency onto colorectal cancers. Oncogene 2020; 39: 3458–3472. 32103169 10.1038/s41388-020-1232-5

[24] Yu J , Yusoff PAM , Woutersen DTJ , *et al*. The functional landscape of patient‐derived RNF43 mutations predicts sensitivity to Wnt inhibition. Cancer Res 2020; 80: 5619–5632. 33067269 10.1158/0008-5472.CAN-20-0957

[25] Seino T , Kawasaki S , Shimokawa M , *et al*. Human pancreatic tumor organoids reveal loss of stem cell niche factor dependence during disease progression. Cell Stem Cell 2018; 22: 454–467.e6. 29337182 10.1016/j.stem.2017.12.009

[26] Miyoshi H , Ajima R , Luo CT , *et al*. Wnt5a potentiates TGF‐β signaling to promote colonic crypt regeneration after tissue injury. Science 2012; 338: 108–113. 22956684 10.1126/science.1223821 PMC3706630

[27] Kawasaki H , Ohama T , Hori M , *et al*. Establishment of mouse intestinal myofibroblast cell lines. World J Gastroenterol 2013; 19: 2629–2637. 23674870 10.3748/wjg.v19.i17.2629 PMC3645381

[28] Yamamoto E , Suzuki H , Yamano H , *et al*. Molecular dissection of premalignant colorectal lesions reveals early onset of the CpG island methylator phenotype. Am J Pathol 2012; 181: 1847–1861. 22995252 10.1016/j.ajpath.2012.08.007

[29] Niinuma T , Kitajima H , Kai M , *et al*. UHRF1 depletion and HDAC inhibition reactivate epigenetically silenced genes in colorectal cancer cells. Clin Epigenetics 2019; 11: 70. 31064417 10.1186/s13148-019-0668-3 PMC6505222

[30] Umar A , Boland CR , Terdiman JP , *et al*. Revised Bethesda Guidelines for hereditary nonpolyposis colorectal cancer (Lynch syndrome) and microsatellite instability. J Natl Cancer Inst 2004; 96: 261–268. 14970275 10.1093/jnci/djh034 PMC2933058

[31] The Cancer Genome Atlas Network . Comprehensive molecular characterization of human colon and rectal cancer. Nature 2012; 487: 330–337. 22810696 10.1038/nature11252 PMC3401966

[32] Parsons R , Myeroff LL , Liu B , *et al*. Microsatellite instability and mutations of the transforming growth factor beta type II receptor gene in colorectal cancer. Cancer Res 1995; 55: 5548–5550. 7585632

[33] Hempen PM , Zhang L , Bansal RK , *et al*. Evidence of selection for clones having genetic inactivation of the activin A type II receptor (*ACVR2*) gene in gastrointestinal cancers. Cancer Res 2003; 63: 994–999. 12615714

[34] Hashimoto T , Yamashita S , Yoshida H , *et al*. WNT pathway gene mutations are associated with the presence of dysplasia in colorectal sessile serrated adenoma/polyps. Am J Surg Pathol 2017; 41: 1188–1197. 28614199 10.1097/PAS.0000000000000877

[35] Tammela T , Sanchez‐Rivera FJ , Cetinbas NM , *et al*. A Wnt‐producing niche drives proliferative potential and progression in lung adenocarcinoma. Nature 2017; 545: 355–359. 28489818 10.1038/nature22334 PMC5903678

[36] Greicius G , Kabiri Z , Sigmundsson K , *et al*. *PDGFRα* ^ *+* ^ pericryptal stromal cells are the critical source of Wnts and RSPO3 for murine intestinal stem cells *in vivo* . Proc Natl Acad Sci U S A 2018; 115: E3173–E3181. 29559533 10.1073/pnas.1713510115 PMC5889626

[37] Donehower LA , Creighton CJ , Schultz N , *et al*. *MLH1*‐silenced and non‐silenced subgroups of hypermutated colorectal carcinomas have distinct mutational landscapes. J Pathol 2013; 229: 99–110. 22899370 10.1002/path.4087 PMC3926301

[38] Oshima H , Oshima M , Kobayashi M , *et al*. Morphological and molecular processes of polyp formation in *Apc*Δ716 knockout mice. Cancer Res 1997; 57: 1644–1649. 9135000

[39] Wu ZQ , Li XY , Hu CY , *et al*. Canonical Wnt signaling regulates Slug activity and links epithelial–mesenchymal transition with epigenetic Breast Cancer 1, Early Onset (BRCA1) repression. Proc Natl Acad Sci U S A 2012; 109: 16654–16659. 23011797 10.1073/pnas.1205822109 PMC3478591

[40] Tenbaum SP , Ordóñez‐Morán P , Puig I , *et al*. β‐catenin confers resistance to PI3K and AKT inhibitors and subverts FOXO3a to promote metastasis in colon cancer. Nat Med 2012; 18: 892–901. 22610277 10.1038/nm.2772

