# Supplementary material for: Characterization of RNF43 frameshift mutations that drive Wnt ligand‐ and R‐spondin‐dependent colon cancer
Source: J Pathol. 2022 Mar 4;257(1):39–52. doi: 10.1002/path.5868 (PMC9314865; doi:10.1002/path.5868)
Supplement: Supplementary file 1 — Supplementary materials and methods [file PATH-257-39-s002.doc]

**Characterization of *RNF43* frameshift mutations that drive Wnt ligand- and R-spondin-dependent colon cancer**

D Yamamoto *et al. J Pathol* DOI: 10.1002/path.5868

**Supplementary materials and methods**

Reference numbers refer to the main text list

*Organoid culture medium*

Established organoid cells were cultured in advanced DMEM/F-12 medium with 10 mm HEPES, 2 mm Glutamax, 1× B27 (Invitrogen, Carlsbad, CA, USA), 1 mm *N*-acetyl-cysteine (Sigma-Aldrich, St Louis, MO, USA), supplemented with 50 ng/ml mouse EGF (#PMG8044, Invitrogen), 10 nm gastrin (#G9145, Sigma-Aldrich), 10 μm SB202190 (#S7067, Sigma-Aldrich), 5 μm A83-01 (#2939; Tocris, Bristol, UK), 10 μm Y-27632 (FUJIFILM Wako Pure Chemicals, Osaka, Japan), and 50% of L-WRN cell-derived conditioned medium (CM) [26].

*Antibodies for immunostaining of tissues*

For immunohistochemistry, antibodies for Ki67 (#ab16667; Abcam, Cambridge, UK), β-catenin (#C2206, Sigma-Aldrich), and active β-catenin (#05-665, Sigma-Aldrich) were used as the primary antibody.

For immunofluorescence (IF), antibodies for GFP (#2956; Cell Signaling Technologies, Danvers, MA, USA) and DsRed (#632392; Clontech, Mountain View, CA, USA) were used to detect Venus and tdTomato-labeled cells, respectively. Alexa Fluor 594- or Alexa Fluor 488-conjugated antibodies (Molecular Probes, Eugene, OR, USA) were used as second layers. DAPI was used for nuclear counterstaining.

*Antibodies for immunocytochemistry*

An antibody for E-cadherin (#AF748; R&D Systems, Minneapolis, MN, USA) was used to detect cell membranes. For the detection of EdU, organoids were stained with a Click-iT EdU Alexa Fluor 488 Imaging Kit (#C10337, Molecular Probes). DAPI was used for nuclear staining.

*Antibodies for immunoblotting*

Antibodies for β-catenin (#C2206, Sigma-Aldrich), active β-catenin (#05-665, Sigma-Aldrich), and GAPDH (#016-25523, FUJIFILM Wako Pure Chemicals) were used. The ECL detection system (GE Healthcare, Buckinghamshire, UK) was used to detect immunoblotting signals.

*PCR primers for amplification of RNF43 fragments (Figure 5B)*

For C14 and C24 For C45

F1: ctgttgggcccttcccggagt F2: tcggtgctgcgcatccggtgc

R1: agagccaggggtgggctcgga R2: ctgggatggcaggaagggacc

*TaqMan PCR primers and probes*

PCR primer-F: gatggcaggaagggacca

PCR primer-R: ccctgctgcctacctgttg

TaqMan MGB probe for wild-type *RNF43* codon 370 (VIC): cggcccccacgacc

TaqMan MGB probe for mutant *RNF43* p.Pro370fs (FAM): tcggccccacgacc

*RT-PCR primers for AXIN2*

F: tggtgccctaccattgacaca

R: tggtcaaccctcaagacctttaaga

*RT-PCR primers for ZNRF3*

F: ctccaggacactcaggagtcca

R: gtctccatacagttcccaatttcca

*Transposon insertion site analysis*

Transposon insertion frequency in *Rnf43*, *Znrf3*, and *Apc*, and insertion sites in *Rnf43* in the intestinal tumors developed in *Trp53^R172H^* mutant mice were examined using data obtained from *Sleeping Beauty* (SB) transposon mutagenesis experiments that were performed previously by Dr Nancy Jenkins’ group [18]. (Dr Haruna Takeda, first author of ref 18, is a co-author of the present study.)
