# Supplementary material for: Characterization of RNF43 frameshift mutations that drive Wnt ligand‐ and R‐spondin‐dependent colon cancer
Source: J Pathol. 2022 Mar 4;257(1):39–52. doi: 10.1002/path.5868 (PMC9314865; doi:10.1002/path.5868)
Supplement: Supplementary file 2 — Figure S1. The results of CIMP analysis for RNF43 frameshift mutant organoid cells Figure S2. Wnt ligand and R‐spondin dependency of RNF43 frameshift mutant organoid cells Figure S3. Biological characteristics of RNF43 frameshift mutant CRC cells Figure S4. Suppression of RNF43 frameshift mutant tumor development by PORCN inhibitor Table S1. Clinicopathological characteristics of CRC patients Table S2. Mutation variants and characteristics of CRC organoids Table S3. Activated upstream regulators in APC‐deleted CRCs compared with RNF43 truncation mutation CRCs (IPA) [file PATH-257-39-s001.doc]

**Characterization of *RNF43* frameshift mutations that drive Wnt ligand- and R-spondin-dependent colon cancer**

D Yamamoto *et al. J Pathol* DOI: 10.1002/path.5868

**Supplementary Figures S1–S4**

**Supplementary Tables S1–S3**

**
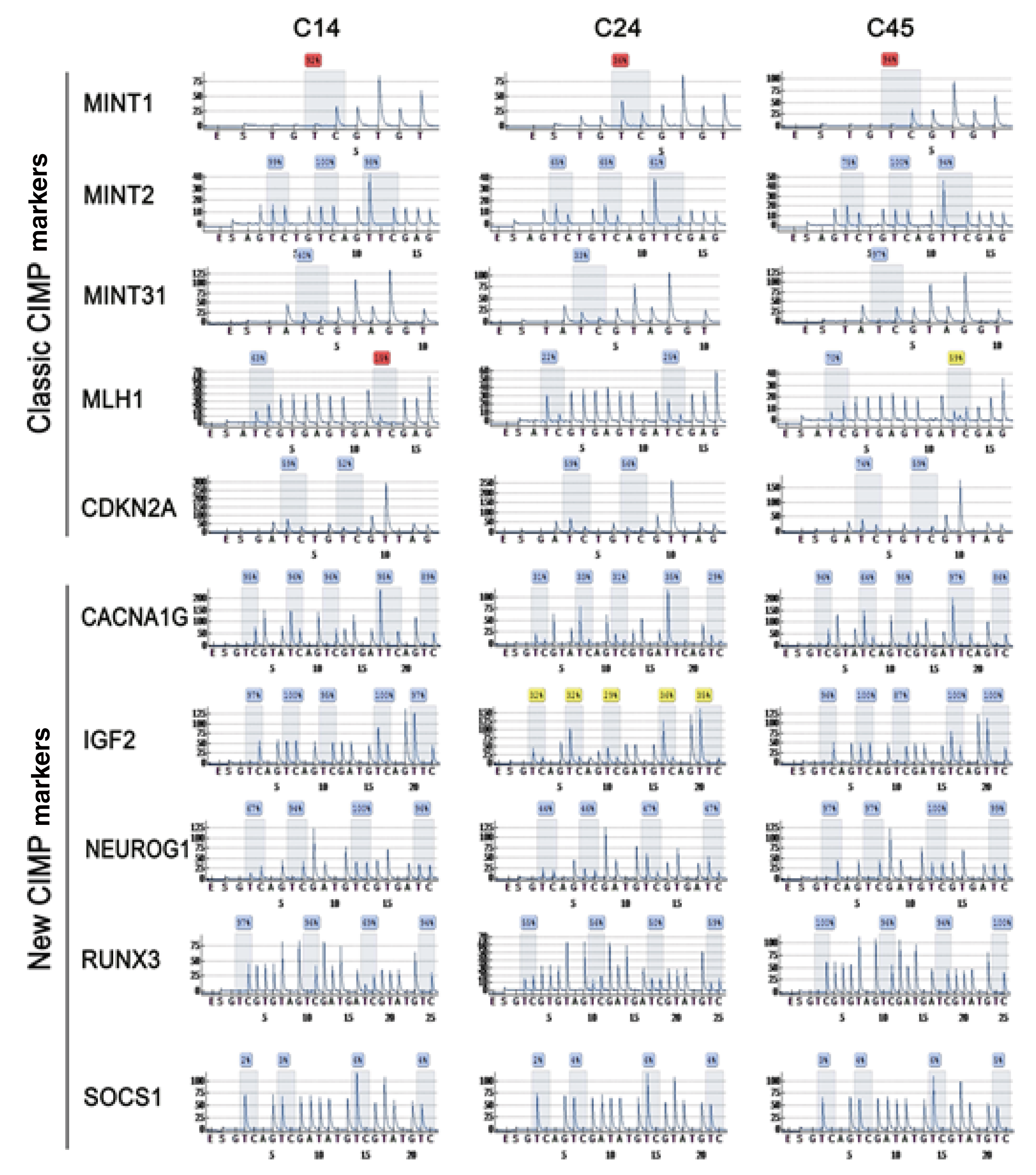
**

**Figure S1.** The results of CIMP analysis for *RNF43* frameshift mutant organoid cells. Representative bisulfite pyrosequencing results for the classic CIMP markers and new CIMP markers are shown.


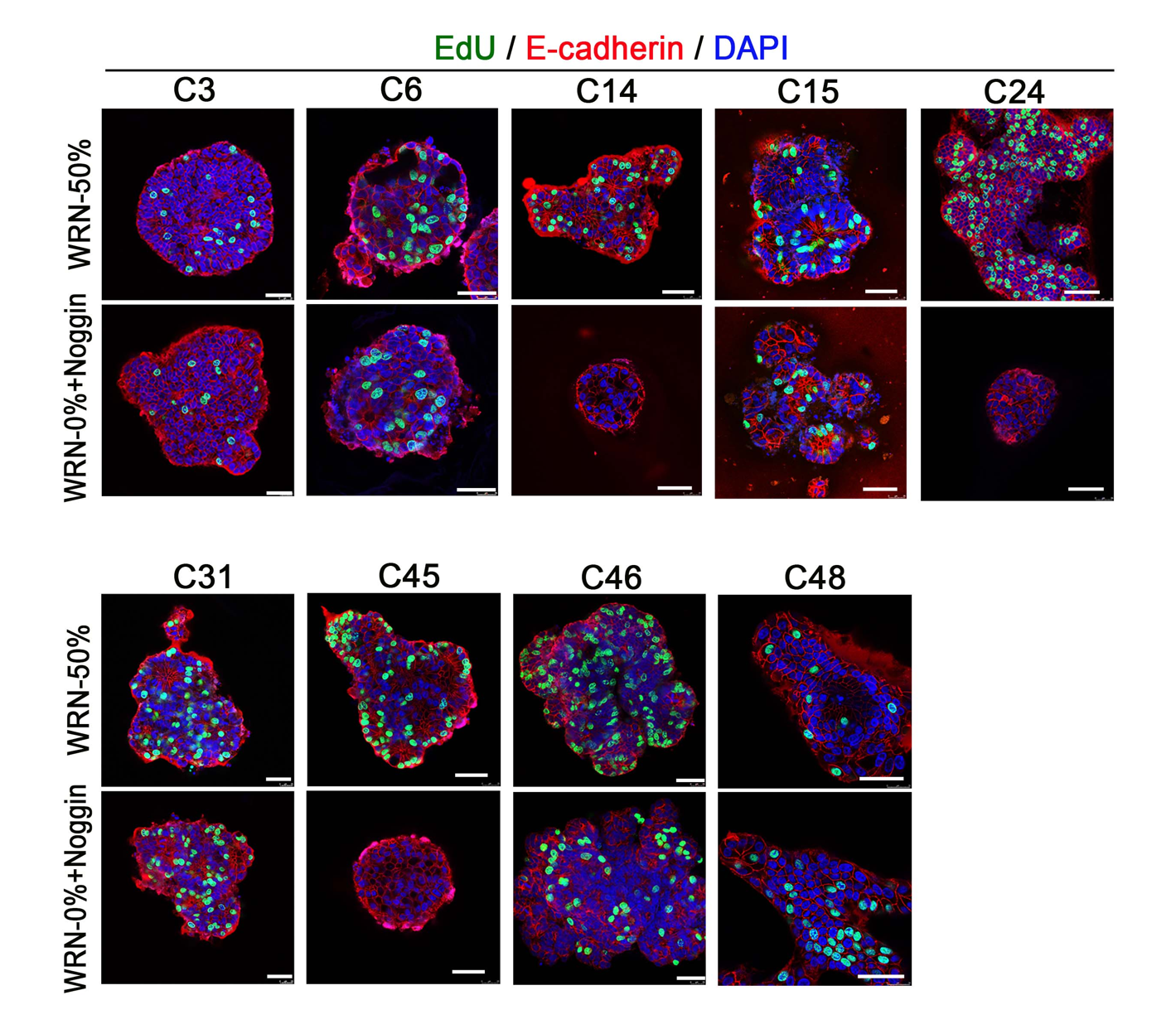


**Figure S2.** Wnt ligand and R-spondin dependency of *RNF43* frameshift mutant organoid cells. Organoids were cultured under WRN-50% (top) and WRN-0% + Noggin (bottom) conditions. Representative fluorescence images of organoids with EdU (green), anti-E-cadherin antibody (red), and DAPI (blue) staining. Note that EdU labeling efficiency was significantly suppressed in C14, C24, and C45 *RNF43* frameshift mutant organoids in the WRN 0% condition. Bars: 50 μm.


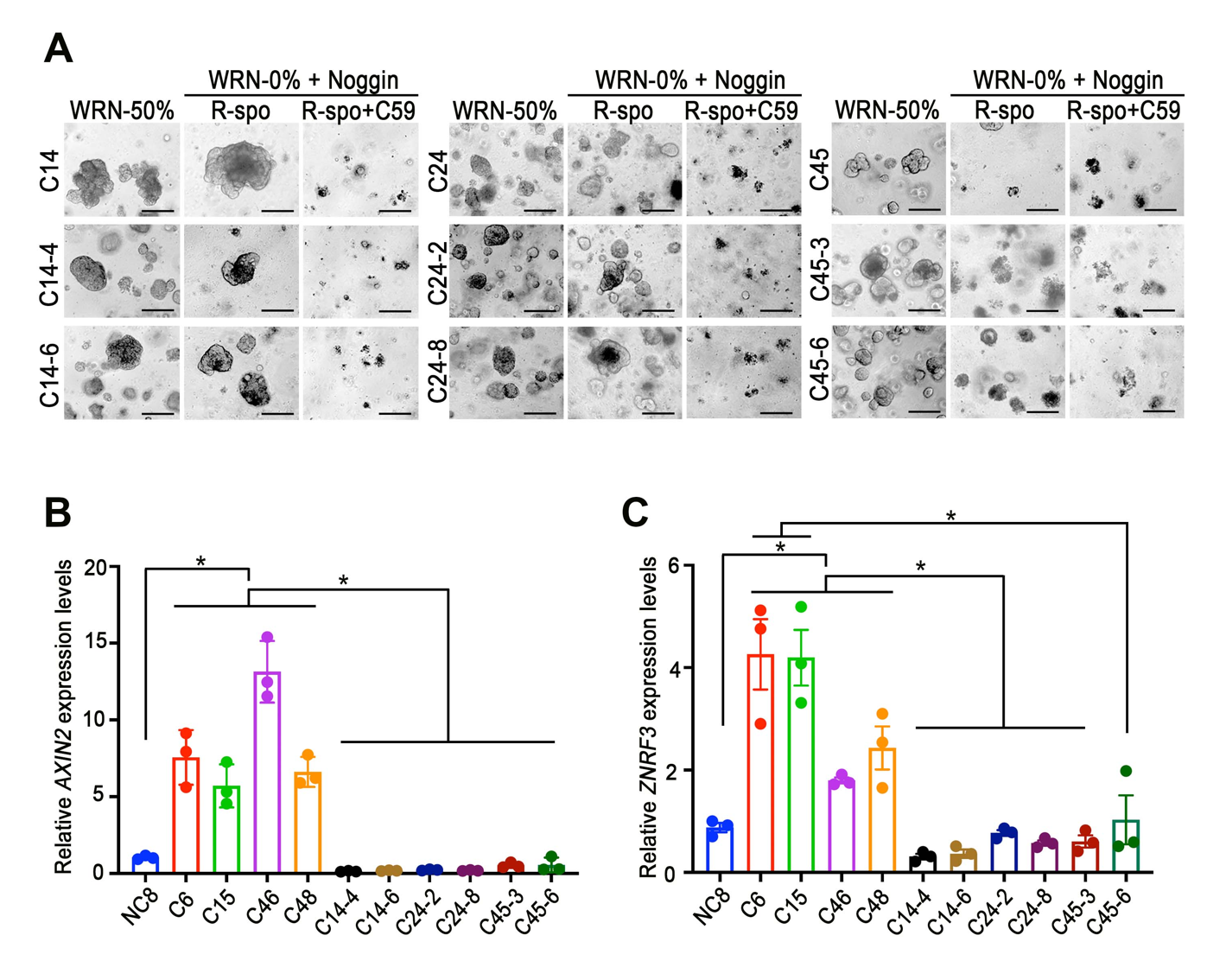


**Figure S3.** Biological characteristics of *RNF43* frameshift mutant CRC cells. (A) Wnt ligand and R-spondin dependency of C14, C24, and C45 parental and subcloned organoids. Representative images of the indicated organoid lines cultured at WRN-50%, WRN-0% + Noggin with R-spondin, or WRN-0% + Noggin with R-spondin and C59. Bars: 250 μm. Note that R-spondin rescued the proliferation of C14, C24, and their subclones under the WRN-0% + Noggin condition, while C45 and the subclones did not proliferate under the same condition. PORCN inhibitor C59 suppressed the proliferation of C14, C24, and their subclones in the WRN-0% + Noggin with R-spondin condition. (B, C) The results of the RT-PCR for (B) *AXIN2* and (C) *ZNRF3* are shown in bar charts with dots (mean ± SD). **p* < 0.05. Note that *AXIN2* and *ZNRF3* expression levels are significantly lower in *RNF43* frameshift mutant CRC cells compared with those in *APC*-mutated conventional pathway-type CRC cells.


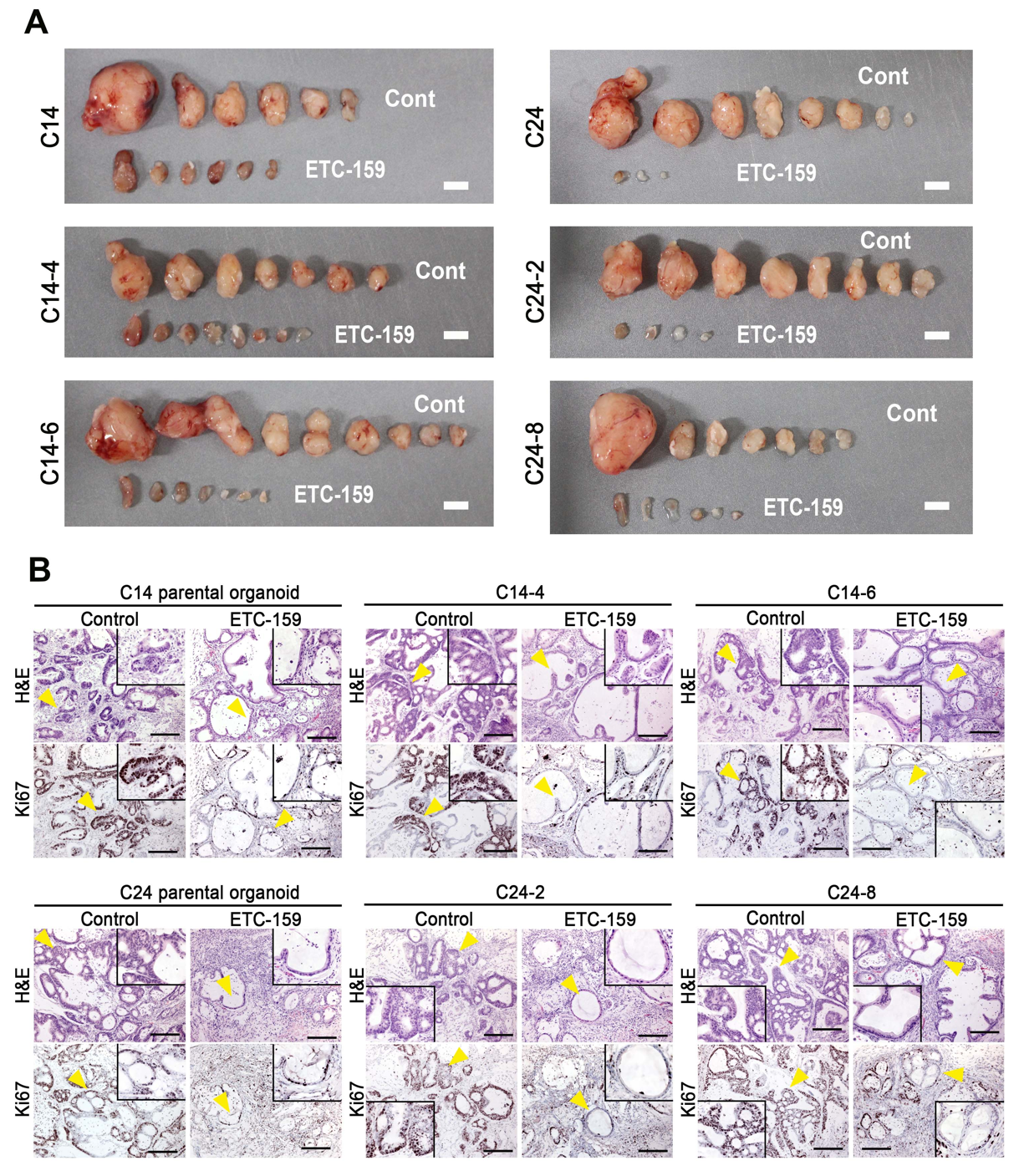


**Figure S4.** Suppression of *RNF43* frameshift mutant tumor development by PORCN inhibitor. (A) Representative photographs of s.c. PDX tumors of C14 and C24 parental organoids and subclones (C14-4, C14-6, C24-2, and C24-8) developed in control mice and ETC-159-treated mice. Bars: 5 mm. (B) Representative histology images of PDX tumors developed in the control and ETC-159-treated mice transplanted with C14 and subclone organoids (top) and C24 and subclone organoids (bottom). H&E (top) and immunohistochemistry for Ki67 (bottom). Insets are enlarged images of the regions indicated by yellow arrowheads. Bars: 200 μm.

**Table S1**. Clinicopathological characteristics of CRC patients

Case ID Sex Age Histological type Location TNM Stage

C3 M 80 Tub1* Distal T3N1M0 IIIB

C6 F 75 Tub1 Distal T3N0M0 IIA

C14 M 80 Tub2^†^ Proximal T3N0M0 IIA

C15 F 58 Tub1 Distal T3N1M0 IIIB

C24 M 74 Tub1 Distal T1aN0M0 I

C31 M 80 Muc^‡^ Distal T3N2M1c IVC

C45 M 69 Tub2 Distal T3N0M0 IIA

C46 M 64 Tub1 Distal T2N0M0 I

C48 F 75 Tub1 Proximal T2N0M0 I

*Well-differentiated tubular adenocarcinoma.

^†^Moderately differentiated tubular adenocarcinoma.

^‡^Mucinous adenocarcinoma.

**Table S2.** Mutation variants and characteristics of CRC organoids

| Organoid lines | C3 | C6 | C14 | C15 | C24 | C31 | C45 | C46 | C48 |
| --- | --- | --- | --- | --- | --- | --- | --- | --- | --- |
| *APC* | p.Ala1184fs | p.Gln1291fs |  | p.Arg876*  p.Thr1493fs | p.Val1326fs | p.Ala1184fs |  | p.Arg232*  p.Glu1322* | p.Gln789*  p.Ser1495fs |
| *RNF43* |  |  | p.Pro370fs  p.Gly659fs |  | p.Pro370fs  p.Gly659fs |  | p.Arg225fs  p.Pro370fs |  |  |
| *KRAS* |  |  |  |  |  |  |  | p.Gly12Asp | p.Gly12Asp |
| *BRAF* |  |  | p.Val600Glu |  | p.Val600Glu |  | p.Val600Glu |  |  |
| *TGFBR2* |  |  | p.Lys153fs  p.Arg553Cys |  | p.Lys153fs  p.Arg553Cys |  | p.Lys153fs  p.Arg553Cys |  |  |
| *ACVR2A* |  |  | p.Phe32fs |  | p.Phe32fs | p.Lys437fs | p.Phe32fs |  |  |
| *TP53* |  | p.Arg196* |  |  | p.Leu137Gln |  |  |  |  |
| Nuclear  β-catenin  (primary)^†^ | + | − | − | + | − | − | − | + | − |
| Nuclear  β-catenin  (PDX)^‡^ | + | − | − | + | − | − | − | + | − |
| Active  β-catenin  (WB)^§^ | High | Low | Low | High | Low | Low | Low | High | High |
| CIMP | NA | NA | High | NA | High | NA | High | NA | NA |
| MSI | − | − | High | − | High | − | High | − | − |

fs, frameshift mutation.

*Amino acid changed to a stop codon.

^†^Nuclear β-catenin accumulation in primary CRC.

^‡^Nuclear β-catenin accumulation in PDX tumor.

^§^Band intensity of β-catenin western blot.

NA, not analyzed.

**Table S3**. Activated upstream regulators in *APC*-deleted CRCs compared with *RNF43* truncation mutation CRCs (IPA)*

| **Upstream regulator** | **Molecule type** | **Predicted activation state** | **Z-score** | ***P* value** |
| --- | --- | --- | --- | --- |
| PPARA | Ligand-dependent nuclear receptor | Activated | 2.908 | 0.00149 |
| CTNNB1^†^ | Transcription regulator | Activated | 2.464 | 1.94E-11 |
| WNT1^†^ | Cytokine | Activated | 2.392 | 0.000499 |
| BMP2 | Growth factor | Activated | 2.384 | 0.0303 |
| Lithium chloride^†^ | Chemical drug | Activated | 2.186 | 0.0101 |
| LARP1 | Translation regulator | Activated | 2 | 0.0116 |

*Activated pathways in *APC* homozygous deleted CRC compared with *RNF43* truncation mutant CRC are listed (Z-score ≥ 2; *p* < 0.05).

^†^Wnt/β-catenin-related pathways are highlighted in yellow.
